# Integrin-linked kinase can facilitate syncytialization and hormonal differentiation of the human trophoblast-derived BeWo cell line

**DOI:** 10.1186/1477-7827-7-51

**Published:** 2009-05-22

**Authors:** Trina M Butler, Pia A Elustondo, Greg E Hannigan, Daniel J MacPhee

**Affiliations:** 1 Division of BioMedical Sciences, Faculty of Medicine, Health Sciences Centre, Memorial University of Newfoundland, St. John's, NL, A1B 3V6, Canada; 2 Centre for Cancer Research, Monash Institute of Medical Research, 246 Clayton Rd., Clayton Melbourne 3168, Australia; 3 Research Institute, Hospital for Sick Children, 555 University Avenue, Toronto ON, M5G 1X8, Canada

## Abstract

**Background:**

In the fusion pathway of trophoblast differentiation, stem villous cytotrophoblast cells proliferate and daughter cells differentiate and fuse with existing syncytiotrophoblast to maintain the multi-nucleated layer. Integrin-linked kinase (ILK) is highly expressed in 1st and 2nd trimester villous cytotrophoblast cells, yet barely detectable in syncytiotrophoblast, thus we examined the potential role of ILK in aiding trophoblast fusion.

**Methods:**

The temporal/spatial expression and activity of ILK were determined in BeWo cells undergoing syncytialization by immunoblot and immunofluorescence analyses. BeWo cells were also transfected with pEGFP expression vectors containing wildtype or two mutant ILK cDNA constructs. The incidence of cell fusion in transfected cells grown under syncytialization conditions was then scored by the presence or absence of E-cadherin immunostaining. Beta-hCG expression in transfected cells, a marker of syncytiotrophoblast hormonal differentiation, was also similarly assessed.

**Results:**

ILK catalytic activity increased and ILK began to increasingly localize to BeWo cell nuclei during syncytialization in correlation with increased pAkt and Snail protein expression. Syncytialization was also significantly elevated (p < 0.05) in BeWo cells expressing constitutively active (ca)-ILK vs cells containing empty vector or dn-ILK. Furthermore, cytoplasmic Beta-hCG expression markedly increased (p < 0.05) in cells expressing wt- and ca-ILK.

**Conclusion:**

ILK-facilitated syncytialization is dependent, at least in part, on ILK catalytic activity while hormonal differentiation appears dependent on both ILK-associated protein interactions and catalytic activity. This study demonstrates that ILK plays a novel role in BeWo syncytialization and differentiation, perhaps through an ILK-Akt-Snail pathway, and implicates ILK in the same process in villous cytotrophoblasts in vivo.

## Background

The human placenta is a critical organ formed during pregnancy that possesses an array of specialized metabolic, hormonal, and immunological functions that control the growth and viability of the fetus and, in turn, the health of the mother [1-4]. The importance of proper placental development to the health and well being of the fetus and mother is illustrated in conditions that arise during pregnancy such as preeclampsia, gestational diabetes and intrauterine growth restriction, that are thought to be the result of placental abnormalities [5].

In the human fetal placenta, the floating villi represent the majority of the chorionic villi and are bathed in maternal blood to function in hormone transport and to aid in the exchange of gases, nutrients and waste between the mother and fetus [2,5]. The floating villi consist of an outer multinucleated syncytiotrophoblast layer, an underlying mitotically active mononuclear cytotrophoblast layer and a stroma [4]. In the cytotrophoblast layer, polarized stem cytotrophoblast cells proliferate and daughter cells then differentiate and fuse with existing syncytiotrophoblast to maintain the multi-nucleated layer [reviewed by [6]]. Morphometric analyses have indicated that in the first trimester there is an excess of villous cytotrophoblast cells fusing with the syncytiotrophoblast – likely necessary for the metabolic integrity of the syncytiotrophoblast [7], although Ellery et al [8] has recently demonstrated that a proportion of nuclei in the syncytiotrophoblast are actively engaged in transcription in accordance with the high metabolic and secretory activity of the tissue. While proliferation of villous cytotrophoblast cells falls with advancing gestation, the cytotrophoblast layer is not entirely discontinuous. Mori et al [9] and Jones et al [10] have calculated ~45–80% continuity of the cell layer at term with cytotrophoblast cells being transformed into flat cells with many thin cellular interdigitating processes. While the events leading to maintenance of the cytotrophoblast stem cell population as well as cytotrophoblast differentiation and fusion are poorly understood, it is becoming clear that initiation of an apoptosis cascade occurs early in differentiation [11,12]. A flip of phosphatidylserine within the cytotrophoblast cell membrane is also associated with cytotrophoblast fusion [7,13]. Since the villous cytotrophoblast and syncytiotrophoblast comprise the epithelial covering of the chorionic villi that is in contact with maternal blood, any disturbances in the processes of cytotrophoblast proliferation and fusion of cytotrophoblast with overlying syncytiotrophoblast can seriously perturb the turnover and function of the syncytiotrophoblast layer and ultimately may contribute to development of fetal growth restriction or preeclampsia [14].

Research has demonstrated that syncytin-1, a retroviral envelope protein, appears to have a direct role in human trophoblast fusion [15]. Furthermore, glial cells missing-1 (GCM1), a transcription factor, appears to be upregulated in pre-fusing cytotrophoblast and to regulate syncytin-1 mRNA expression [15-17]. Along with these findings, other proteins or groups of proteins such as protein tyrosine kinases and protein tyrosine phosphatases with a variety of other known functions also appear to play important roles in the fusion pathway [18,19]. An intracellular serine/threonine kinase named Integrin-linked kinase (ILK) localizes to focal adhesions and is critically involved in the adhesion of cells to their extracelluar environment and in signal transduction [20,21]. ILK interacts with the cytoplasmic domains of β-integrins and numerous cytoskeletal associated proteins and has been found to mediate protein-protein interactions.

Recently, Elustondo et al [22] reported that ILK was highly detectable by immunoblot analyses in human chorionic villous tissue lysates throughout gestation. It was also highly expressed *in situ *in villous cytotrophoblast cells and in stromal mesenchyme in first trimester and early second trimester human chorionic floating villi; however, it was scarcely detected in the syncytiotrophoblast layer. The adherens junction protein E-cadherin mediates homophilic calcium- dependent cell adhesion in neighbouring cells and is also highly detectable at points of cytotrophoblast cell-cell contact [23,24]. E-cadherin expression markedly decreases with remodeling of cell-cell adhesion complexes associated with differentiation and subsequent fusion of cytotrophoblast to syncytiotrophoblast [24] and, thus, presence/absence of the protein can be used to assess trophoblast syncytialization [25-27]. As yet, the mechanism(s) underlying the downregulation of E-cadherin expression in villous cytotrophoblast undergoing the morphogenetic process of syncytialization is unknown.

Hannigan et al [28] has demonstrated that over-expression of ILK in epithelial cells results in disrupted cell-cell adhesion. ILK also downregulates E-cadherin expression through activation of the transcriptional repressor Snail, independent of β-catenin/T cell factor (TCF7) regulation [29]. Based on the reported role of ILK in regulating E-cadherin expression and the known expression of both ILK and E-cadherin expression in the cytotrophoblast of human chorionic villi during pregnancy, we hypothesized that ILK could be a player in regulating the differentiation and fusion of cytotrophoblast into the syncytiotrophoblast via the downregulation of E-cadherin. To begin testing this hypothesis we employed a well documented cell line model, BeWo cytotrophoblast cells, for the study of trophoblast syncytialization [30,31].

## Methods

### Cell culture

The BeWo cell line was purchased from the American Type Culture Collection (Cat. #CCL-98, Manassas, VA, USA). This cell line was originally derived from a human trophoblast choriocarcinoma and is well known to undergo syncytialization upon forskolin treatment [32]. This cell line model is ideal for experiments because syncytialization can be controlled with syncytialization of up to 80% [33]. They can be grown under proliferating conditions indefinitely where they exhibit an epithelial phenotype or under syncytialization conditions where it is well known that BeWo cells express markers of syncytiotrophoblast such as β-hCG, syncytin, and markedly decreased E-cadherin and desmoplakin expression [15,24,33-36].

Cells were cultivated in 75 cm^2 ^culture flasks in Ham's F-12 media with L-glutamine (Cat. #11765-062; Invitrogen Ltd., Burlington, Ontario, Canada) supplemented with 10% fetal bovine serum (Cat. #16000-044; Invitrogen) and 100 U penicillin/100 μg (Cat. #15140-122; Invitrogen) as has been described elsewhere [37]. The BeWo cells were maintained under standard culture conditions of 5% carbon dioxide in air at 37°C with medium renewal on a daily basis. This tissue culture media formulation and associated culture parameters were designated "proliferating conditions". Once cells reached ~80% confluency they were passaged into new tissue culture flasks with new media at a ratio of 1:4. For culture of BeWo cells to promote syncytialization the same conditions as above were utilized except that the media was simply changed to Ham's F-12K media (Cat. # 21127-022) containing 25 μM Forskolin (Cat. #F6886; Sigma Chemical Co, St. Louis, Missouri, USA). This media formulation and culture parameters were designated as "syncytialization conditions".

### BeWo transfection and determination of syncytialization and β-hCG expression

BeWo cells were counted with a haemocytometer and the concentration was adjusted to 2.5 × 10^5 ^cells/ml. Cells were initially seeded on either 22 × 22 cm glass coverslips, seated in 35 mm tissue culture dishes, or in 6 well plates and cultivated in proliferating conditions as described above. When the cells reached approximately 80% confluence they were washed with media without FBS or antibiotics and transiently transfected with 2.0 μg of empty pEGFP-C3 vector (Cat. #6082-1; BD Clontech), pEGFP-C3 containing human wild-type (wt)-ILK, pEGFP-C3 containing dominant-negative human E395K (dn)-ILK, or pEGFP-C3 containing a constitutively active human S343D (ca)-ILK construct and 8.0 μl of Lipofectamine 2000 (Cat. #11668-027; Invitrogen). Transfections were conducted according to the manufacturer's detailed instructions. Six hours after transfection the cells were cultivated in syncytialization conditions and the media replenished 24 hours after transfection.

The incidence of syncytialization in transfected cells was scored by the presence or absence of E-cadherin immunostaining in twenty microscopic high power fields of view (400× observed magnification). The use of E-cadherin immunostaining has been utilized in the past for such assessments of trophoblast fusion [25-27]. Syncytialization was considered genuine when at least 3 or more nuclei were present in the same cytoplasm. The incidence of β-hCG expression in transfected cells was also scored by the presence or absence of β-hCG immunostaining in twenty microscopic high power fields of view (400× observed magnification).

### Immunocytochemical analysis

#### BeWo cell syncytialization time course

BeWo cells were initially seeded on 22 × 22 mm glass coverslips (2.5 × 10^5 ^cells/coverslip) seated in 35 mm culture dishes and grown in proliferating conditions for 24 hours. Cells were then fixed in 4% paraformaldehyde/PBS for 15 minutes under these proliferating conditions (0 h) or subsequently at 3, 6, 9, 12, 18, 24, 36, or 48 h after initiation of culture in syncytialization conditions. Cells were then washed once with 1 × PBS for 5 minutes.

#### ILK fusion protein overexpression

For immunolocalization of E-cadherin or β-hCG in EGFP-fusion protein expressing cells, forty eight hours after transfection BeWo cells were washed with 1 × PBS for 2 × 5 minutes each. The cells were then fixed in 4% paraformadehyde/PBS for 15 minutes and then washed once with 1 × PBS for 5 minutes.

For immunocytochemical analyses, all cells were placed in PBS containing 0.1% Triton X-100 (PBT) for 15 minutes at room temperature and subsequently washed with 1 × PBS for 5 minutes. The cells were then blocked in 5% normal goat serum/1% horse serum/1% fetal bovine serum in PBS for 1 h at room temperature with constant agitation, then incubated for 1 hour at room temperature in appropriate primary antisera (Table 1). Affinity-purified mouse and rabbit IgG, at the same concentration as the primary antisera, served as negative controls for immunocytochemical analyses. After three washes in PBT, cells were incubated with appropriate secondary antisera (Table 1). The cells were then washed two times with PBT followed by a final wash in PBS. The cells were then mounted in Vectashield containing DAPI (Cat # H-1200; Vector Laboratories Inc., Burlington, Ontario, Canada). All cells were observed using a Leica DM-IRE2 inverted microscope (Leica Microsystems, Richmond Hill, Ontario, Canada) equipped for epifluorescence illumination and attached to a Retiga Exi CCD camera (QImaging, Burnaby, B.C, Canada). Openlab Image Analysis software (Version 5.5; Improvision, Inc., Lexington, Massachusetts, USA) was used for image capture and analysis.

**Table 1:** Antisera utilized for immunofluorescence and immunoblot analysis.

Antisera	Method	Dilution	Company	Catalogue #
Mouse anti-ILK; Clone 65.1.9	IF	1:100	Abcam, Ltd, Cambridge, MA, USA	ab49979
Rabbit anti-ILK	IB	1:2000	Cell Signaling Technology, Beverly, MA, USA	3862
Rabbit anti-E-cadherin	IF	1:250	Abcam, Ltd, Cambridge, MA, USA	ab15148
Mouse anti-E-cadherin, clone SHE78-7	IF IB	1:1000 1:10000	EMD Biosciences, San Diego, CA, USA	205602
Rabbit anti-hCG	IF	1:500	Dako Canada, Inc, Mississauga, ON, Canada	A0231
Rabbit anti-Snail	IB	1:1000	Abcam, Ltd, Cambridge, MA, USA	ab17732
Mouse anti-pAKT1 (Ser 473); Clone 587F11	IF	1:100	Cell Signaling Technology, Beverly, MA, USA	4051
Rabbit anti-pAKT1 (Ser 473)	IB	1:1000	Cell Signaling Technology, Beverly, MA, USA	9271
Anti-phospho-GSK3β (Ser-9)	IB	1:1000	Cell Signaling Technology, Beverly, MA, USA	9336
FITC-Sheep anti-Rabbit IgG	IF	1:250	Sigma Chemical Co, St. Louis, MO, USA	F7512
RRX-Donkey anti-Mouse IgG	IF	1:150	Jackson ImmunoResearch Labs Inc, West Grove, USA	715-295-150
RRX-Donkey anti-Rabbit IgG	IF	1:250	Jackson ImmunoResearch Labs Inc, West Grove, USA	711-295-152
ChromPure Mouse IgG	IF	N/A*	Jackson ImmunoResearch Labs Inc, West Grove, USA	015-000-003
ChromPure Rabbit IgG	IF	N/A*	Jackson ImmunoResearch Labs Inc, West Grove, USA	011-000-003
HRP-Goat anti-Rabbit IgG (H+L)	IB	1:20000	Pierce, Rockford, IL, USA	31460
HRP-Goat anti-Mouse IgG (H+L)	IB	1:10000	Pierce, Rockford, IL, USA	31430

IF: immunofluorescence, IB: immunoblot, FITC: Fluorescein isothiocyanate, RRX: Rhodamine-Red-X, HRP: horseradish peroxidase, *Dependent on concentration of primary antisera utilized

### Immunoblot analysis

#### BeWo cell syncytialization time course

BeWo cells were initially seeded at 2.5 × 10^5 ^cells/well in 35 mm culture dishes in Ham's F12 media and cultured for 24 hours. Cells were then lysed from these culture conditions (0 h) or at 3, 6, 9, 12 18, 24, 36, or 48 h after initiation of culture in syncytialization conditions. 250 μl of NP-40 lysis buffer (50 mM Tris pH 8.0, 150 mM NaCl, 1% Nonidet P-40) containing 100 μM sodium orthovanadate and complete Mini EDTA-free protease inhibitors (Roche Molecular Biochemicals, Laval, Quebec, Canada) were added to the cells. Cells were harvested with a plastic cell scraper and homogenized with moderate pipetting. Samples were cleared by centrifugation and supernatants were retained for immunoblot analysis.

#### ILK fusion protein overexpression

BeWo cells were initially seeded at 3.5 × 10^5 ^cells/well in 6 well plates and transiently transfected with either the pEGFP -C3 vector or one of the pEGFP-C3 containing ILK constructs described above. Cells were then cultivated in syncytialization conditions at 6 and 24 hours post-transfection as described above. Forty- eight hours after transfection, 250 μl of NP-40 lysis buffer (50 mM Tris pH 8.0, 150 mM NaCl, 1% Nonidet P-40) containing 100 μM sodium orthovanadate and complete Mini EDTA-free protease inhibitors (Cat. # 11836170001; Roche Molecular Biochemicals, Laval, Quebec, Canada) were added to the cells. Cells were harvested with a plastic cell scraper and homogenized with moderate pipetting. Samples were cleared by centrifugation and supernatants were retained for immunoblot analysis.

For all immunoblot analyses, sample protein concentrations were determined by the Bradford Assay using the Bio-Rad protein assay dye reagent (Bio-Rad Laboratories, Mississauga, Ontario, Canada). Protein samples (30 μg/lane) were separated in 10% polyacrylamide gels under denaturing conditions and gels were blotted to Pierce 0.45 μm nitrocellulose membranes (MJS Biolynx, Inc, ON, Canada). Blots were washed with Tris buffered saline-Tween-20 (TBST; 20 mM Tris base, 137 mM NaCl, and 0.1% Tween 20, pH 7.6) and blocked with 5% BSA/TBST for 1 h. Appropriate antisera (Table 1) were incubated with blots at 4°C overnight with constant agitation. The next day, blots were washed with TBST and then incubated in relevant horseradish peroxidase (HRP)-conjugated goat secondary antisera for 1 hour at room temperature with constant agitation. Following washes in TBST, proteins were detected on immunoblots using the Pierce SuperSignal West Pico chemiluminescent substrate detection system (Cat #34080; MJS Biolynx, Inc) and multiple exposures were generated to ensure the linearity of the film responses.

### β-hCG ELISA

BeWo cells were either grown under proliferating or syncytialization conditions for 48 hours, with the media being replenished once at 24 hours after initial seeding. After 48 hours, the media was collected and the ELISA conducted with a β-hCG ELISA kit (Cat # EIA-1469; DRG Diagnostics, New Jersey, USA) exactly according to the manufacturer's instructions.

### Nonradioactive IP kinase assay

BeWo cells were seeded in 35 mm culture dishes at 2.5 × 10^5 ^cells/well and cultured in either proliferating conditions or syncytialization conditions as described above. After 48 hours of culture, the cells were lysed with NP-40 lysis buffer containing 100 μM sodium orthovanadate and complete Mini EDTA-free protease inhibitors and protein concentrations determined as described previously. For immunoprecipitation, 4.0 μg of mouse monoclonal ILK antisera or non-specific IgG (see Table 1) was added to 400 μg of appropriate BeWo cell lysate and incubated overnight at 4°C with gentle agitation. Fifty microlitres of TrueBlot anti-mouse IP beads (Cat # 00-8811; eBiosciences, San Diego, California, USA) were added to each sample and incubated for 2 hours at 4°C with gentle rocking. Then ILK-antibody-bead complexes were centrifuged at 13,000 × g for 1 minute at 4°C and subsequently washed 3 × with 500 μl of NP- 40 lysis buffer followed by 3 washes with 500 μl of kinase buffer (Cat # 9802; Cell Signaling). Complexes were then each suspended in 50 μl of kinase buffer supplemented with 1.0 μl of 10 mM ATP (Cat # 9804; Cell Signaling) and 1 μg of GSK-3β fusion protein (Cat # 9237; Cell Signaling) and incubated for 30 minutes at 30°C. The reaction was then terminated with 12.0 μl of 5× SDS polyacrylamide gel loading buffer followed by vortexing and centrifugation for 30 seconds at 14,000 × g. All samples were heated to 95°C for 5 minutes prior to loading on 10% polyacrylamide gels and SDS-PAGE followed by electroblotting to Pierce 0.45 μm pore nitrocellulose membrane (MJS Biolynx, Inc). Immunoblot analyses of phosphorylated GSK-3β (Ser-9) and ILK proteins were then conducted.

### Data analysis

Statistical analysis was performed with GraphPad Prism^® ^version 5.01 (GraphPad Software, San Diego, California, USA). Statistical significance for the determination of the incidence of cell fusion and intracellular β-hCG expression were analyzed with a one-way analysis of variance (ANOVA) and a Newman-Keuls multiple comparisons test. Values were considered significantly different if p < 0.05. Densitometric analyses of immunoblot data was conducted with a one-way ANOVA followed by pair wise comparison of data points with two tailed unpaired t-tests.

## Results

### ILK expression during BeWo syncytialization

**Figure 1:**
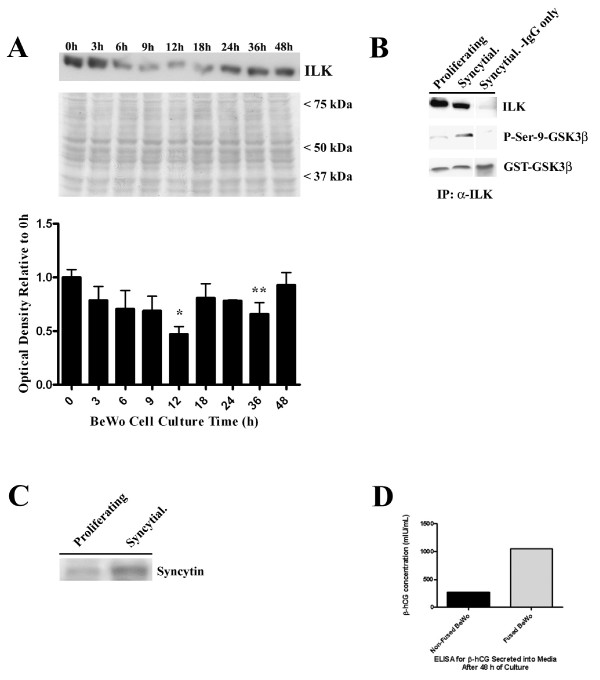
**ILK protein expression and catalytic activity during BeWo syncytialization**. A) Immunoblot analyses of ILK protein expression during a timecourse (0 h – 48 h) of BeWo syncytialization. A representative immunoblot is shown from four independent experiments (n = 4). Middle panel is a representative immunoblot stained for total protein demonstrating comparable protein loading between gel lanes. 37 kDa – 75 kDa represent the position of molecular weight markers. The graph demonstrates the densitometric analysis of ILK protein expression during the timecourse (n = 4). * p < 0.05 vs 0 h and 48 h; **p < 0.05 vs 0 h. B) Representative immunoblots from *in vitro *kinase assays (n = 4) of ILK catalytic activity in BeWo cells grown under proliferating or syncytialization conditions (Syncytial.). Syncytial. -IgG only = immunoprecipitations with non-specific IgG in place of the primary ILK antisera. P-Ser-9-GSK3β = phosphorylated GST-GSK3β fusion protein. GST-GSK3β = total fusion protein used in the assay. C) A representative immunoblot demonstrating increased syncytin protein expression after 48 h of BeWo cell culture in syncytialization conditions (Syncytial.) compared to proliferating conditions (0 h). D) A representative ELISA demonstrating increased β-hCG secretion from BeWo cells after 48 h of culture in syncytialization conditions (Fused BeWo) compared to cell culture under proliferating (Non-fused BeWo) conditions (n = 8).

ILK protein was readily detectable in BeWo cell lysates from cells cultured under proliferating conditions (Fig. 1A; 0 h). Upon culture in syncytialization-promoting conditions of Ham's F12K + 25 um Forskolin, ILK expression consistently decreased by 6 h of culture and was significantly lower by 12 h vs 0 h (p < 0.05). Expression quickly recovered and reached detection levels at 48 h of culture that were comparable to ILK expression at 0 h. In contrast, ILK kinase activity, measured by phosphorylation of a GSK3β fusion protein, was markedly higher at 48 h of culture in syncytialization-promoting conditions compared to activity in cells cultivated in proliferating conditions (Fig. 1B). This result was also concomitant with increased syncytin protein expression and β-hCG secretion occurring in syncytializing BeWo cells, as has been previously described [35,36], compared to cells cultivated under proliferating conditions (Fig. 1C, D). These results thus indicate that ILK protein expression and activity are dynamically regulated during BeWo syncytialization.

**Figure 2:**
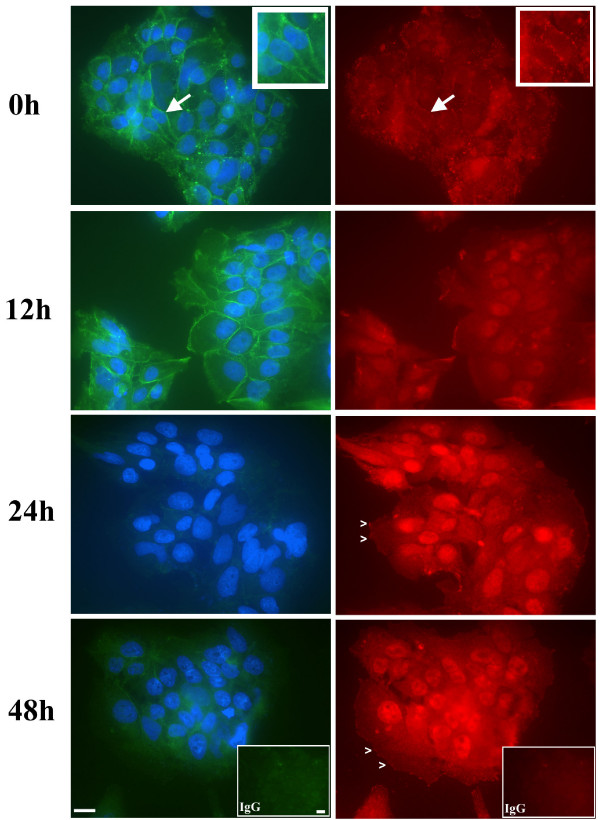
**Immunofluorescence analyses for E-cadherin (left panels) and ILK (right panels) protein expression in BeWo cells during a timecourse of syncytialization**. Cells were cultivated under proliferating conditions (0 h) or syncytialization conditions (12 h – 48 h). With increasing culture time in syncytialization conditions, ILK began to localize to cell nuclei in addition to the cytoplasm and to focal adhesions (arrowheads, 24 h – 48 h). Arrows in 0 h panels show the position of apparent ILK and E-cadherin co-localization demonstrated in the insets. IgG insets, non-specific IgGs of the appropriate animal species were used as specificity controls in place of the appropriate primary antisera. Nuclei in E-cadherin immunofluorescence panels were stained with DAPI. Scale bars = 25 μm.

**Figure 3:**
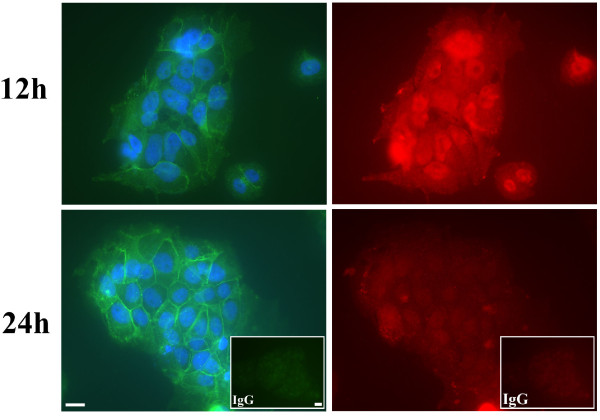
**Immunofluorescence analyses for E-cadherin (left panels) and ILK (right panels) protein expression in BeWo cells at 12 h and 24 h of cell culture in syncytialization conditions**. The images demonstrate that ILK localization to cell nuclei correlates with downregulation of E-cadherin expression at cell-cell membranes. IgG insets, non-specific immunoglobulins of the appropriate animal species were used as specificity controls in place of the appropriate primary antisera. Nuclei in E-cadherin immunofluorescence panels were stained with DAPI. Scale bars = 25 μm.

The spatial localization of ILK also changed dynamically during BeWo syncytialization (Fig. 2). Under proliferating culture conditions (0 h), ILK was localized in the cell cytoplasm, at focal adhesion-like structures and particularly present at cell-cell adhesions marked by E-cadherin expression (Fig. 2, arrows; inset). Once BeWo cells were cultured under syncytialization conditions, ILK localization shifted over the timecourse of culture to an increased nuclear localization concomitant with decreasing E-cadherin detection in cell-cell adhesions (Fig. 2). However, ILK could still be detected at decreased levels in the cytoplasm at 12 h and then in the cytoplasm and to focal-adhesion like structures at the cell periphery from 24–48 h (Fig. 2). Since BeWo cells grown under syncytialization conditions can achieve up to 80% syncytialization [33], some BeWo cell clusters do not syncytialize. Interestingly, in these cases we consistently observed that where syncytialization was not evident, as marked by sustained and readily detectable E-cadherin in cell-cell adhesions, ILK localization to cell nuclei was markedly decreased (Fig. 3; 24 h vs 12 h panels).

### Consequences of ILK fusion protein expression in BeWo cells

**Figure 4:**
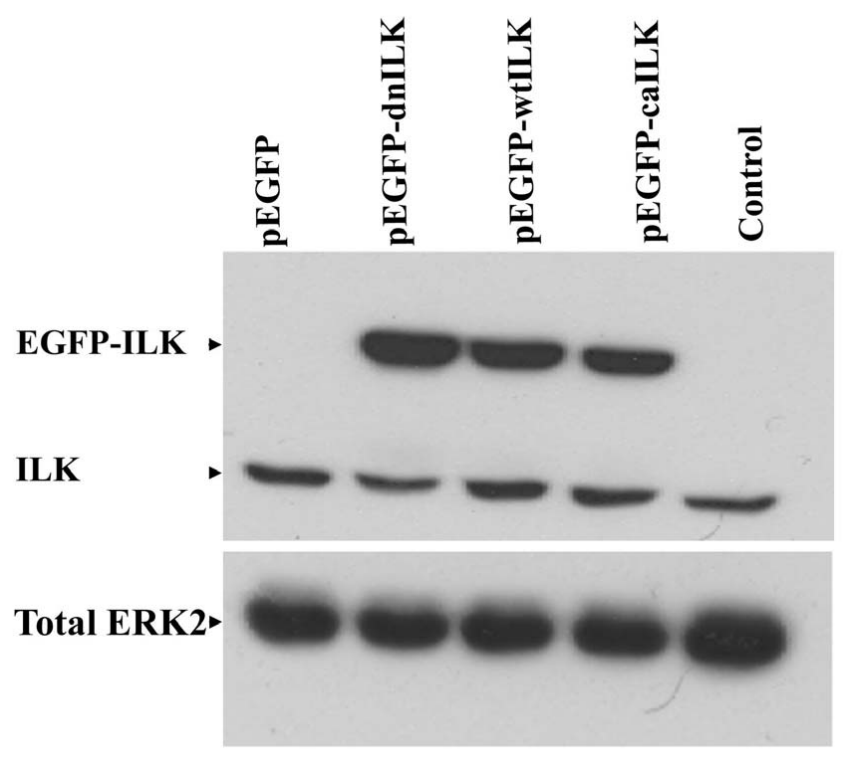
**Immunoblot analysis of transient expression of wildtype (wt), dominant negative (dn), and constitutively active (ca) EGFP-ILK fusion proteins in BeWo cells**. A representative immunoblot demonstrating that the EGFP-ILK fusion proteins were readily detectable in BeWo cells 48 h post-transfection and absent in the empty vector transfected cells (pEGFP). Endogenous ILK (ILK) was detectable in all transfected and non-transfected cell lysates and total ERK2 detection served as an indication of comparable protein loading between lanes.

BeWo cells were transfected under proliferating conditions with pEGFP-C3 expression vectors containing wildtype or mutant ILK cDNAs. Cells were then grown under fusion promoting conditions to assess the impacts of the expression of various EGFP-ILK proteins on trophoblast fusion. Transient expression of EGFP-wt-ILK, EGFP-dn-ILK, and EGFP-ca-ILK for 48 h in BeWo cells under fusion conditions resulted in significant detection of these proteins on immunoblots at the expected ~80 kDa, in addition to endogenous levels of ILK (Fig. 4). These results confirmed the viability of these vectors and also demonstrated that the expression of the various EGFP-ILK proteins were comparable between the respective transfectants for our studies. The expression of all the EGFP-fusion proteins was also readily observable by immunofluorescence.

**Figure 5:**
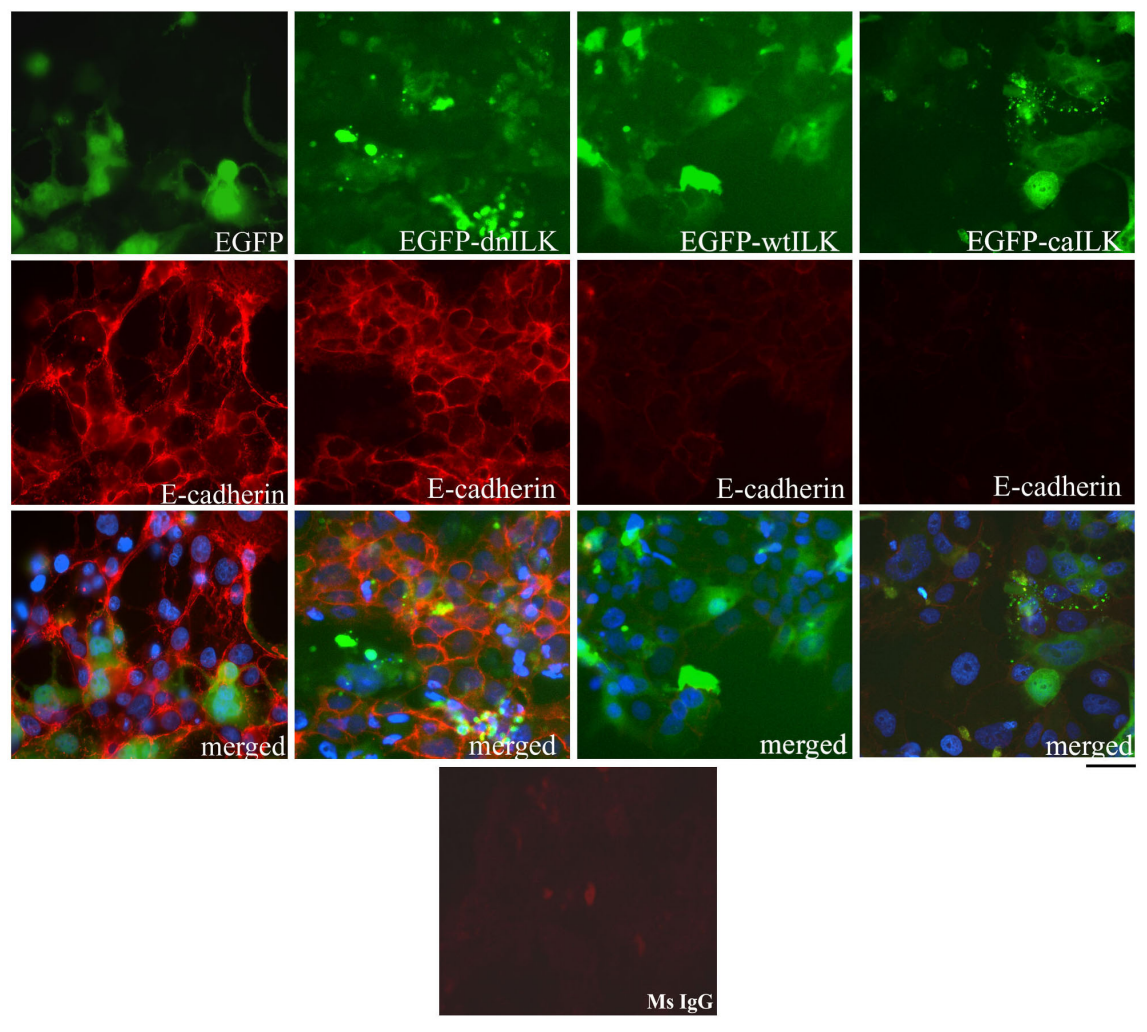
**Immunocytochemical analysis of E-cadherin expression in BeWo cells transiently transfected with either pEGFP-C3 vector (EGFP), pEGFP-C3 containing dominant negative ILK (EGFP-dnILK), wildtype ILK (EGFP-wt ILK) or constitutively active ILK (EGFP-ca ILK) and cultivated under syncytialization conditions for 48 h**. Ms IgG = mouse IgG negative control. Scale bar = 50 μm. Data are representative of three independent experiments.

During culture of BeWo cells under both proliferating and syncytialization conditions, cells were routinely monitored by phase contrast microscopy to qualitatively assess the condition of the cell cultures. To determine the incidence of syncytialization in transfected cells, the presence (no syncytialization) or absence (syncytialization) of E-cadherin in cell membranes of EGFP-fusion protein expressing cells was assayed by immunofluorescence analysis (Fig. 5). Transient expression of dn-ILK in BeWo cells resulted in a slight decrease in trophoblast cell fusion, compared to vector control transfected cells, but the decreased levels did not reach statistical significance (Fig. 5; Table 2). In contrast, expression of wt-ILK caused a significant increase in cell fusion, compared to dn-ILK expressing cells (p < 0.05), and expression of ca-ILK in BeWo cells significantly increased cell fusion (P < 0.05) compared to dn-ILK and empty vector expressing cells (Fig. 5; Table 2).

**Table 2:** The incidence of BeWo syncytialization. The incidence of BeWo syncytialization was determined by scoring the absence of E-cadherin immunolocalization in groups of EGFP-fusion protein expressing cells. Data are from three independent experiments.

Experiment	BeWo + pEGFP-C3	BeWo + pEGFP-C3 -dn-ILK	BeWo + pEGFP-C3 - wt-ILK	BeWo + pEGFP-C3- ca-ILK
1	28/43	11/38	35/48	38/43
2	26/51	39/63	66/72	32/35
3	21/50	14/47	19/32	27/37
				
Avg. Incidence (% of 1.00 +/- SEM)	0.52 +/- 0.07	0.42 +/- 0.10	^a^0.73 +/- 0.09	^b^0.85 +/- 0.06

a: Syncytialization was significantly elevated in cells expressing wildtype (wt)-ILK versus dominant negative (dn)-ILK expressing cells (p < 0.05)

b: Syncytialization was significantly increased in BeWo cells expressing constitutively active (ca)-ILK vs cells containing pEGFP-C3 (empty vector) or dn-ILK (p < 0.05)

**Figure 6:**
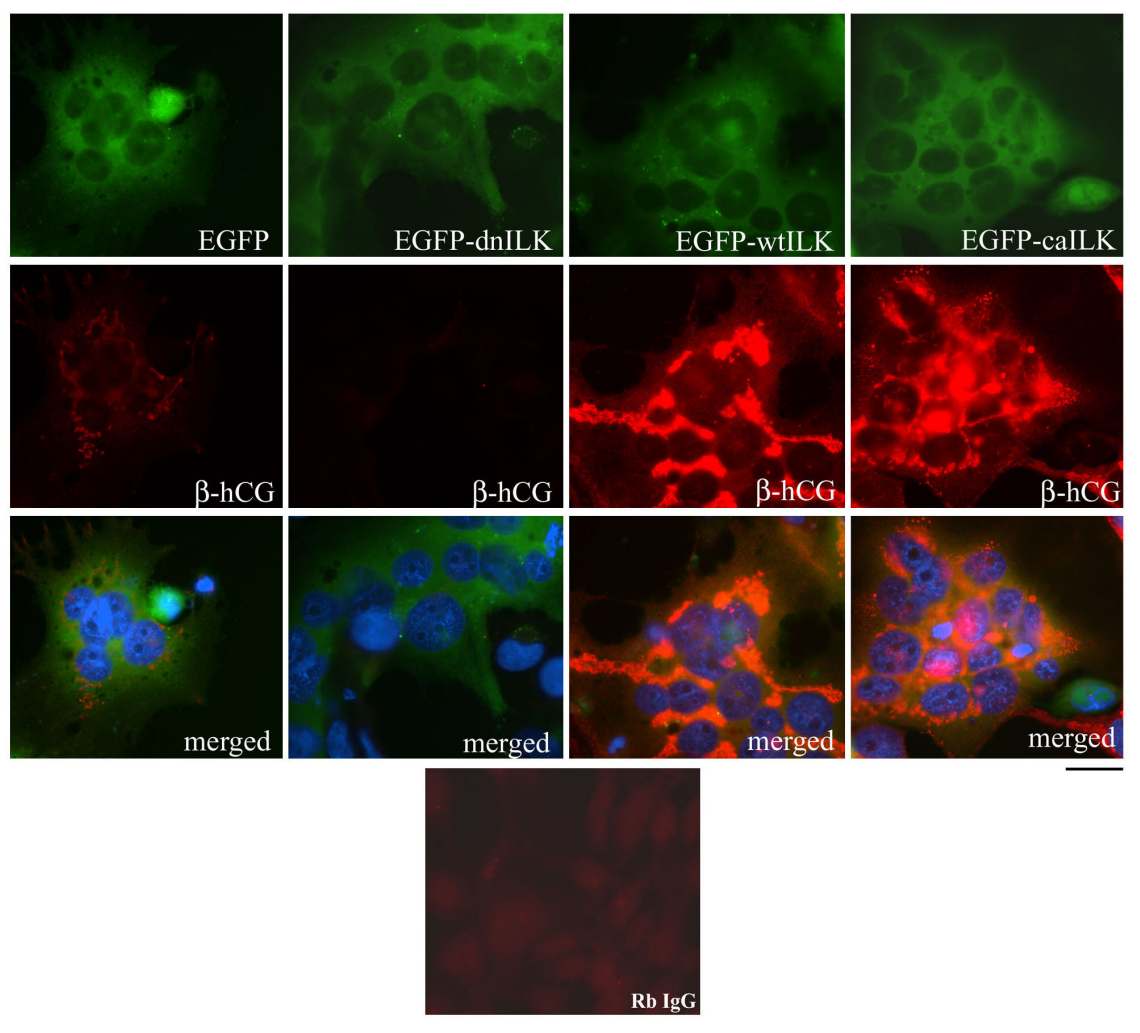
**Immunocytochemical analysis of β-human chorionic gonadotropin (β-hCG) expression in BeWo cells transiently transfected with pEGFP-C3 vector (EGFP), pEGFP-C3 containing dominant negative ILK (EGFP-dn ILK), wildtype ILK (EGFP-wt ILK) or constitutively active ILK (EGFP-ca ILK) and cultivated under syncytialization conditions for 48 h**. Rb IgG = rabbit IgG negative control. Scale bar = 25 μm. Data are representative of three independent experiments.

Upon stimulation of BeWo cells with forskolin, it is well known that β-hCG expression and secretion are upregulated [35], thus serving as a marker of trophoblast hormonal differentiation. ELISA analyses of β-hCG secretion into culture media from transiently transfected BeWo cells demonstrated that any differences in β-hCG secretion could not be statistically distinguished between the different transfected cells (data not shown) likely due to the background of β-hCG secretion from non-transfected BeWo cells. Thus, using immunofluorescence analysis we specifically examined the cytoplasmic expression of β-hCG in transfected BeWo cells (Fig. 6). BeWo cells transfected with empty vector did express some β-hCG in the cytoplasm but dn-ILK expressing cells showed dramatically reduced (p < 0.05) β-hCG expression (Fig. 6; Table 3). In contrast, wt-ILK and ca-ILK expressing cells showed markedly enhanced expression (p < 0.05) of β-hCG in the cell cytoplasm compared to dn-ILK and vector control cells (Fig. 6; Table 3).

**Table 3:** The incidence of β-human chorionic gonadotropin (β-hCG) expression. The incidence of β-hCG expression was determined by the presence of β-hCG immunolocalization in groups of EGFP-fusion protein expressing cells. Data are from three independent experiments.

Experiment	BeWo + pEGFP-C3	BeWo + pEGFP-C3- dn-ILK	BeWo + pEGFP-C3- wt-ILK	BeWo + pEGFP-C3- ca-ILK
1	16/40	13/68	27/47	46/57
2	23/57	8/51	30/47	49/61
3	20/43	15/55	22/36	38/55
				
Avg. Incidence (% of 1.00 +/- SEM)	0.43 +/- 0.02	^a^0.22 +/- 0.03	^b^0.62 +/- 0.02	^b, c^0.77 +/- 0.04

a: Cytoplasmic β-hCG expression markedly decreased in cells expressing dominant negative (dn)-ILK relative to pEGFP-C3 (empty vector) cells (p < 0.05)

b: Expression was significantly increased in cells expressing wildtype (wt)- and constitutively active (ca)-ILK compared to cells expressing empty vector or dn-ILK (p < 0.05)

c: Cytoplasmic β-hCG expression was significantly increased in cells expressing ca-ILK compared to cells expressing wt-ILK (p < 0.05)

### Expression of candidate ILK partners in BeWo syncytialization

**Figure 7:**
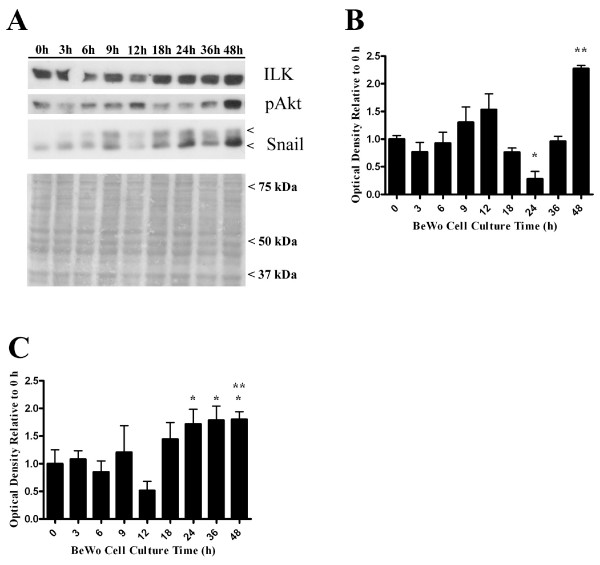
**ILK, pAkt, and Snail protein expression during a timecourse of BeWo syncytialization**. A) Representative immunoblots from four independent experiments (n = 4) are shown with a representative immunoblot stained for total protein to illustrate comparable protein loading of all gel lanes. B) Densitometric analysis of pAkt expression during BeWo cell culture in syncytialization conditions demonstrating a significant decrease in pAkt expression by 24 h (p < 0.05) compared to 0, 9, 12 and 48 h and a rapid increase in pAkt expression by 48 h of culture compared to 0 h (p < 0.05, n = 4). C) Densitometric analysis of Snail protein expression during BeWo cell culture in syncytialization conditions demonstrating that Snail expression becomes significantly elevated at 24, 36 and 48 h vs 12 h (p < 0.05, n = 4) and at 48 h of culture compared to expression in proliferating conditions at 0 h (p < 0.05, n = 4).

To begin identifying candidate molecules that could partner with ILK to promote syncytialization, we examined a timecourse of BeWo cell syncytialization by immunoblot analysis. Phosphorylated (Ser 473)-Akt expression, a substrate of ILK activity, consistently increased by 48 h of BeWo culture under syncytialization-promoting conditions (Fig. 7A, B; 0 h vs 48 h, p < 0.05), in support of our data from in vitro kinase assays of ILK activity at 48 h of culture (Fig 1B). Furthermore, the expression of the transcriptional repressor Snail was relatively low at 0 h until after 12 h of culture in syncytialization conditions then also became highly expressed by 48 h compared to 0 h (Fig. 7A, C; p < 0.05). Thus, increased ILK activity appears to correlate with increased Snail expression during syncytialization and decreased detection of E-cadherin at cell-cell adhesions (Figs. 1, 2, 7).

## Discussion

Despite well known roles for ILK in cell-ECM adhesion and signal transduction [20,21], ILK also appears to be involved in integrin-independent epithelial cell-cell adhesion [38,39]. We have previously reported that ILK was highly expressed in villous cytotrophoblast cells in first trimester and early second trimester human chorionic floating villi, but that it was barely detected in the syncytiotrophoblast layer [22]. The adherens junction protein E-cadherin is also highly detectable in human cytotrophoblast and loss of E-cadherin in cytotrophoblast is associated with syncytialization [24-27]. Importantly, ILK appears to regulate E-cadherin expression [28,29]. Thus, using the BeWo trophoblast cell line model we examined ILK expression in BeWo cells during syncytialization and tested the hypothesis that ILK could facilitate the differentiation and fusion of cytotrophoblast into the syncytiotrophoblast.

### Temporal and spatial expression of ILK is dynamically altered during syncytialization

Examination of ILK kinase activity during syncytialization revealed that ILK activity is increased during syncytialization. This could lead to increased ILK-Akt signaling as Akt is a substrate of ILK. While total ILK expression was significantly lower by 12 h (vs 0 h) of culture in syncytialization conditions, expression quickly recovered and reached detection levels at 48 h of culture that were comparable to ILK expression at 0 h. However, more dramatic changes in spatial expression of ILK were apparent during syncytialization as ILK began accumulating in cell nuclei. Recent work by Acconcia et al [40] demonstrated that ILK can localize to both cell cytoplasm and cell nuclei and that ILK contains both functional nuclear localization and nuclear export sequences. Furthermore, in MCF7 and NIH 3T3 cells ILK nuclear localization appears important for nuclear integrity and ILK can associate with chromatin. Since changes in ILK expression and catalytic activity were associated with BeWo syncytialization, we examined whether or not exogenous ILK overexpression in BeWo cells could facilitate the process.

### ILK facilitates BeWo syncytialization

The examination of the presence or absence of desmosomal or adherens junction (E-cadherin) proteins has been successfully used for evaluating the incidence of fusion in trophoblast [18,24,26,41,42]. Thus, we also scored the incidence of syncytium formation in BeWo cells overexpressing EGFP-ILK fusion proteins by examining the presence or absence of E-cadherin immunolocalization. Transient expression of wt-ILK in BeWo cells significantly increased trophoblast syncytialization compared to dn-ILK while ca-ILK expression significantly increased syncytialization compared to vector control and dn-ILK expressing BeWo cells. The ILK constructs used for our experiments have been utilized by other laboratories, including ours, in a number of cell types [20,21]. We have previously demonstrated that transient expression of dn-ILK in the extravillous trophoblast cell line model HTR8-SVneo significantly reduced trophoblast migration compared to cells expressing wt-ILK [22]. The over-expression of wt-ILK in a number of cell types can lead to phosphorylation of Akt and GSK3-β while the active mutant ca-ILK has been shown to constitutively phosphorylate Akt [43-45]. The dominant negative mutant ILK was originally considered a kinase dead mutant, but has since been shown to have ~20% kinase activity in vitro [45-47] be deficient in α-parvin and paxillin interaction, and to be incapable of incorporating into focal adhesions thus remaining only in the cytoplasm [48,49]. Therefore, it appears to exert a strong dominant negative effect by maintaining ILK associated proteins in an inappropriate subcellular location (e.g. not focal adhesions) [49].

Since dn-ILK expressing BeWo cells only displayed a slightly reduced level of syncytialization compared to vector control cells, it would appear that lack of focal adhesion incorporation of ILK-associated proteins, but not likely α-parvin or paxillin, does not significantly impact BeWo syncytialization. Furthermore, the likelihood of residual kinase activity from this mutant (not fully kinase dead) may account for the less than significant reduction in syncytialization in dn-ILK expressing BeWo cells. Transient expression of wt-ILK in BeWo cells did not significantly increase trophoblast syncytialization compared to vector control expressing cells. ILK activity is dependent on PI-3 kinase activity in vivo [21] and, as a result, it is possible that the overexpression of the wt-ILK fusion protein may have saturated the endogenous PI-3 kinase resulting in low activation levels of the exogenous wt-ILK fusion protein. In contrast, our results with ca-ILK expressing BeWo cells indicate that ILK facilitates BeWo trophoblast syncytialization and that it is likely dependent, at least in part, on ILK catalytic activity. Fusion-promoting culture conditions, particularly elevated cAMP levels and PKA activation induced by forskolin addition [32,50], also likely synergize with ILK catalytic activity to aid syncytialization as ILK-induced syncytialization was absent in BeWo cells grown only under proliferation-promoting conditions (data not shown). In total, our results clearly correlate with work by Miller et al [51] who demonstrated that over-expression of ILK in L6 myoblasts resulted in increased ILK activity and stimulation of myoblast fusion into myotubes.

### ILK induces intracellular β-hCG expression

The synthesis and secretion of β-hCG is a marker of syncytiotrophoblast differentiation [6] and syncytializing BeWo cells also express β-hCG mRNA and protein when cultivated under fusion-promoting conditions [15,34,35]. In our experiments we examined β-hCG expression in transfected cells using immunocytochemistry, as has been previously done [34], since our transient transfection strategy meant there was a background of β-hCG expression and secretion from untransfected cells. In vector control cells we detected some β-hCG expression as a result of culture in fusion-promoting conditions; however, dn-ILK expressing cells exhibited significantly lower levels of β-hCG expression in situ compared to vector controls and the other mutant ILK protein expressing cells. Thus, in contrast to syncytialization, lack of proper subcellular localization of ILK-associated proteins (dominant negative effect) does impact BeWo cell β-hCG expression and differentiation. Furthermore, with the concern above regarding proper catalytic activation of exogenous wt-ILK in transfected BeWo cells, the significant increase in β-hCG expression in these cells vs dn-ILK and vector controls may also indicate a need for ILK, in addition to catalytic activity, as an adapter protein and signaling platform for other signalling proteins to aid proper hormonal differentiation. What specific ILK-associated protein(s) may be involved in hormonal differentiation of BeWo cells is under investigation. The significant upregulation of β-hCG expression in ca-ILK expressing BeWo cells highlights that ILK catalytic activity, at least in part, also has a role in promoting syncytiotrophoblast differentiation.

### Candidate ILK signalling pathway

The underlying signaling mechanism that could be responsible for ILK facilitated syncytialization of BeWo cells may involve Snail, a sensitive transcriptional repressor of E-cadherin expression. Tan et al [29] have previously demonstrated that over-expression of ILK in human colon carcinoma cell lines stimulated Snail expression. Recently, these results were extended in Scp2 mouse mammary epithelial cells where ILK over-expression resulted in stimulation of Snail expression and loss of E-cadherin expression [52]. Alternatively, depletion of ILK, Akt, or Snail resulted in upregulation of E-cadherin expression. The authors also identified Poly (ADP-ribose) polymerase-1 (PARP-1) as a component of the signaling pathway upstream of Snail leading to downregulation of E-cadherin expression [52]. Our results clearly establish a correlation of increased ILK activity and alterations in ILK expression, including nuclear localization of ILK, during BeWo syncytialization with increased Snail expression in these cells during this process. With reports of Snail involved in promoting epithelial-mesenchymal transition by downregulating E-cadherin expression through an ILK-Akt-Snail pathway [29,52], our study also implicates this pathway in facilitating syncytialization of BeWo trophoblast cells. Identification of additional members of this ILK signaling pathway and the specific interactions and role(s) of all these proteins in trophoblast syncytialization is ongoing.

Impaired trophoblast fusion and differentiation appears to be directly associated with pathological conditions such as preeclampsia or fetal growth restriction [14]. Using DNA microarray analysis, Kudo et al [35] reported that genes involved in cell and tissue structural dynamics appeared to be very important for syncytialization. Given the role of ILK in such processes, we appear to have identified a new protein involved in trophoblast syncytialization and differentiation for future study. In addition, our research also raises the possibility of a more general role for ILK in cell-cell fusion *per se *as this fusion process has been suspected in breast cancer cells and demonstrated in macrophages and myoblasts – and all of which can highly express ILK [51,53-56].

## Conclusion

These findings demonstrate that ILK activity and expression play a novel role in syncytialization and differentiation of BeWo cells, and implicate ILK in the same process in villous cytotrophoblasts in vivo.

## Competing interests

The authors declare that they have no competing interests.

## Authors' contributions

TB carried out the experiments and contributed to the writing of the manuscript. PE was involved in the cultivation of the BeWo. GH contributed the expression vectors that were constructed and tested in his laboratory. DJM conceived and designed the study, assisted with statistical analyses and assisted with drafting of the manuscript. All authors read and approved the final manuscript.
